# Heterodimerization of the prostaglandin E2 receptor EP2 and the calcitonin receptor CTR

**DOI:** 10.1371/journal.pone.0187711

**Published:** 2017-11-02

**Authors:** Shin Matsubara, Akira Shiraishi, Tsubasa Sakai, Toshimi Okuda, Honoo Satake

**Affiliations:** Bioorganic Research Institute, Suntory Foundation for Life Sciences, Kyoto, Japan; Indian Institute of Technology Kanpur, INDIA

## Abstract

G protein-coupled receptors (GPCRs) have been found to form heterodimers and modulate or fine-tune the functions of GPCRs. However, the involvement of GPCR heterodimerization and its functional consequences in gonadal tissues, including granulosa cells, have been poorly investigated, mainly due to the lack of efficient method for identification of novel GPCR heterodimers. In this paper, we identified a novel GPCR heterodimer between prostaglandin E2 (PGE2) receptor 2 (EP2) and calcitonin (CT) receptor (CTR). High-resolution liquid chromatography (LC)-tandem mass spectrometry (MS/MS) of protease-digested EP2-coimmunoprecipitates detected protein fragments of CTR in an ovarian granulosa cell line, OV3121. Western blotting of EP2- and CTR-coimmunoprecipitates detected a specific band for EP2-CTR heterodimer. Specific heterodimerization between EP2 and CTR was also observed by fluorescence resonance energy transfer analysis in HEK293MSR cells expressing cyan- and yellow-fluorescent protein-fused EP2 and CTR, respectively. Collectively, these results provided evidence for heterodimerization between EP2 and CTR. Moreover, Ca^2+^ mobilization by CT was approximately 40% less potent in HEK293MSR cells expressing an EP2-CTR heterodimer, whereas cAMP production by EP2 or CT was not significantly altered compared with cells expressing EP2- or CTR alone. These functional analyses verified that CTR-mediated Ca^2+^ mobilization is specifically decreased via heterodimerization with EP2. Altogether, the present study suggests that a novel GPCR heterodimer, EP2-CTR, is involved in some functional regulation, and paves the way for investigation of novel biological roles of CTR and EP2 in various tissues.

## Introduction

Most receptors of neurotransmitters, neuropeptides, and hormones are G protein-coupled receptors (GPCRs), and their pharmacological properties are targets of drug development [1]. GPCRs are not only present as monomers and homodimers but form heterodimers with other GPCRs [2, 3]. GPCR heterodimerization has been found to alter ligand binding affinity, signal transduction, and desensitization of GPCRs [2, 3] and to participate in pathological processes [4, 5], pharmacological profiles [6–8] and species-specific biological events [9] *in vitro* and *in vivo*. Moreover, several orphan GPCRs have been shown to serve as protomers (i.e. partner GPCRs) in GPCR heterodimers and to cause specific functional alterations [10–13]. These findings have revealed the significance of GPCR heterodimers in various biological activities.

Ovarian functions are coordinated and multi-step biological events functionally regulated by a wide range of neuropeptides and hormones. These complicated processes are also thought to be controlled by fine-tuning or functional alterations of GPCR heterodimers. To date, several receptors for reproductive hormones have been found to form GPCR heterodimers. For instance, two major gonadotropin-receptors, follicle-stimulating hormone receptor and luteinizing hormone receptor, have been shown to form heterodimers, which reciprocally attenuate the downstream signaling of each receptor, although an endogenous function of it in ovarian cells remains to be elucidated [14]. Our previous study also demonstrated heterodimerization of the *Ciona intestinalis* gonadotropin-releasing hormone (GnRH) receptor (Ci-GnRHR)4 with Ci-GnRHR1 and Ci-GnRHR2 in vitellogenic follicles [11, 12]. These findings suggested that various GPCR heterodimers participate in a wide range of biological functions in ovaries.

Prostaglandin E2 (PGE2) is a multifunctional lipid in the follicle, and participates in ovulatory processes and fertilization mainly through its cognate GPCR, EP2 that is expressed in granulosa cells [15–18]. Granulosa cells, located between an oocyte and theca cells, are believed to play pivotal roles of oocyte and follicle growth and maturation by endogenous ligands. Indeed, a few transcriptomic analyses detected the expression of various GPCRs in granulosa cells [19, 20]. Collectively, these findings lead to the hypothesis that heterodimerization of EP2 with other GPCRs is involved in the regulation of ovarian and follicular functions. However, little is known about the heterodimerization of EP2 in any tissues or organs including granulosa cells.

The greatest difficulty in studying GPCR heterodimers is the lack of methodologies that can predict novel GPCR heterodimers. A GPCR heterodimerization network was constructed based on experimental data and the overall topology of GPCR heterodimers [21]. Moreover, several computational models and simulations of GPCR heterodimer structures have been designed [22]. Nevertheless, these predictions have not yet led to the identification of novel GPCR heterodimers. In addition, no gene-silencing or knockdown procedures are useful for evaluating the biological effects of GPCR heterodimerization, given that not only a heterodimeric (oligomeric) GPCR but also a monomeric GPCR are downregulated by these methods. Consequently, exploration of novel GPCR heterodimers still depends on conventional experiments, including detection of the co-expression of two GPCRs and the functional relationship between two GPCR overexpressed in cultured cells. These technical issues strongly suggest the need for efficient methods to detect novel GPCR heterodimers.

Non-GPCR membrane protein complexes have been identified by coimmunoprecipitation (Co-IP)-based liquid chromatography (LC)-tandem mass spectrometry (MS/MS) [23, 24]. Although no GPCR heterodimer has yet been identified using this method [23, 24], these findings suggest that Co-IP-based LC-MS/MS may be used to screen for novel GPCR heterodimers. Hence, we aim to develop a Co-IP-based MS procedure and to identify a novel GPCR heterodimer using it. This study presents the identification of a novel GPCR heterodimer between the EP2 and the calcitonin (CT) receptor (CTR) in cultured cell lines using high-resolution LC-MS/MS of EP2-coimmunoprecipitates, and fluorescent resonance energy transfer (FRET). Moreover, we show that heterodimerization of CTR with EP2 functionally altered CT-induced intracellular Ca^2+^ mobilization by CTR.

## Materials and methods

### Cell culture

OV3121 cells, derived from mouse granulosa cells, were the kind gift of Dr. Atsushi P. Kimura of Hokkaido University and were cultured in DMEM supplemented with 10% FBS. HEK293MSR cells were maintained at 37°C under 5% CO_2_ as previously described [11]. After being washed with PBS, cells were collected and centrifuged. The resultant pellets were stored at -80°C until use.

### RT-PCR analysis

Total RNA was extracted and purified using Sepasol-RNA I Super G (Nacalai Tesque, Kyoto, Japan), treated with TURBO DNase I (Ambion, Austin, USA), and reverse-transcribed to the cDNA using oligo(dT)_20_ and Superscript III (Thermo Fisher Scientific, Waltham, USA). PCR was performed as previously described [25] using the primers listed in S1 Table.

### Membrane preparation and Western blotting

Cell pellets were homogenized using a Polytron homogenizer in homogenizing buffer (HB, 10 mM Tris-HCl, 1 mM EDTA, 10 mM MgCl_2_, 11% sucrose, pH 8.0) containing 1 × complete protease inhibitor cocktail (Roche Diagnostics, Mannheim, Germany). After sonication, the homogenate was incubated with 250 μg/ml RNase A (Qiagen, Valencia, USA) on ice for 30 min, releasing ribosomal proteins from the membrane fraction, as described [26]. The homogenate was centrifuged twice at 8,000 × g at 4°C for 10 min each, and then, the supernatant was further centrifuged at 100,000 × g at 4°C for 1 hr. The precipitate was washed, resuspended, sonicated in HB, and solubilized by adding an equal volume of 2 × radioimmunoprecipitation assay buffer (RIPA, 50 mM Tris-HCl, 300 mM NaCl, 2% NP-40, 2% sodium deoxycholate (SDC), 0.2% sodium dodecyl sulfate (SDS)) containing 2 × protease inhibitor cocktail and rotating at 4°C for 1 hr. Aliquots of 15-μg membrane proteins were electrophoresed on polyacrylamide gels and transferred to nitrocellulose membranes. The blot was incubated with 1 μg of each rabbit monoclonal anti-EP2 (ab167171) or anti-CTR (ab11042) antibody (Abcam, Tokyo, Japan) as a primary antibody. The normal rabbit IgG (sc-2027, Santa Cruz Biotechnology, Santa Cruz, USA) and anti-Na^+^/K^+^ ATPase α1 antibody (ab7671, Abcam) were used as a negative and a loading control of membrane proteins, respetively. After the incubation with horseradish peroxidase-linked secondary antibody (NA9340V, GE healthcare, Buckinghamshire, UK), signals were detected using an ECL system (GE healthcare) as previously described [11].

### Co-IP, SDS-PAGE, and in-gel protein digestion

Aliquots of 800-μg membrane proteins were precleared with protein G sepharose (GE healthcare) at 4°C for 1 hr with rotation and centrifuged at 10,000 × g for 1 min. The supernatant was incubated overnight at 4°C with 6-μg anti-EP2 antibody and protein G sepharose. The mixture was centrifuged at 4°C at 10,000 × g for 1 min, and the beads were washed seven times with 1 × RIPA buffer and once with 0.1 × RIPA buffer. To denature proteins, the beads were incubated twice with 1.5 × sample buffer (2.5% SDS, 12.5% glycerol, 0.075% bromophenol blue, 2.5% β-mercaptoethanol) at 37°C for 5 min each. Collected samples were subjected to SDS-PAGE and subsequent fragmentation. Another Co-IP followed by Western blotting was performed to verify the interaction between EP2 and CTR. Aliquots of 30-μg membrane proteins were co-immunoprecipitated with 1 μg of each anti-EP2 or anti-CTR antibody, and one-tenth volume of collected samples (and 3 μg of input sample without Co-IP) were used for Western blotting using anti-CTR or anti-EP2 antibody, respectively. The sample prepared without Co-IP was used as a negative control.

Following SDS-PAGE and staining with Coomassie brilliant blue, the gel fragment corresponding to proteins of molecular mass 25–150 kDa was excised, cut into 1-mm cubes, and dehydrated with 100% acetonitrile (ACN) for 10 min. After drying *in vacuo*, the gel pieces were swollen in a minimum volume of digestion buffer (25 mM NH_4_HCO_3_, 0.1% SDC, 20 ng/μl trypsin/Lys-C mix) for 15 min, covered with digestion buffer, and incubated overnight at 37°C. Gel pieces were washed with MilliQ water and combined with the original supernatant. Proteolytic fragments were extracted three times from gel pieces by mixing in 50% ACN for 1 hr each. All extracts were treated with 0.5% trifluoroacetic acid, followed by centrifugation at 15,700 × g for 2 min. After drying the supernatants *in vacuo*, the residues were dissolved in MilliQ water and purified with YM-3 Microcon filters (Millipore, Bedford, USA).

### LC-MS/MS analysis

The purified protein fragments were subjected to reverse-phase nano-liquid chromatography using an EASY-nLC 1000 system, followed by tandem mass spectrometry (LC-MS/MS) analysis using Orbitrap Elite (Thermo Fisher Scientific). The protein fragment solutions were first loaded onto a 75 μm × 2 cm C18 trap column (Acclaim PepMap100, 3 μm, 100Å) at 300 nl/min and separated on a 50 μm × 15 cm C18 analytical column (Acclaim PepMap RSLC, 2 μm, 100Å) using a linear gradient of 15–30% ACN with 0.1% formic acid over 48 min. The LC eluent was introduced into the mass spectrometer by positive-mode nanospray ionization. Full-scan mass spectra over a range of 350–2,000 *m/z* were measured with Orbitrap at a resolution of 30,000, and the 15 most intense multivalent precursor ions were selected for data-dependent scanning. The MS/MS spectra were acquired with Velos Pro under conditions of 1 *m/z* isolation width, 35% normalized collision energy, and 10 ms maximum ion accumulation time. Data were analyzed with Xcalibur software 2.2.

### Protein identification

Raw files were processed using Proteome Discoverer 1.1 software incorporating the SEQUEST search algorithm. All protein sequences of *Mus musculus* were downloaded from RefSeq (December 2013) [27] and used for protein identification. Search criteria included precursor mass tolerance, 10 ppm; fragment mass tolerance, 1.2 Da; maximum missed cleavage site, 2; and dynamic modification, oxidation of methionine.

### Plasmid construction and transfection

Open reading frames (ORFs) of EP2, CTR, and oxytocin receptor (OXTR) were amplified from the OV3121 by RT-PCR. The ORF of EP2 was subcloned in frame into 5’-end of the pcDNA4/V5 (Thermo Fisher Scientific) and pAmCyan1-N1 cyan fluorescent protein (CFP) vector (Clontech, Kyoto, Japan) at the *Eco*RI/*Xho*I and *Nhe*I/*Xho*I, sites, respectively. The CTR was subcloned into the pcDNA4/V5 and pZsYellow1-N1 yellow fluorescent protein (YFP) vector (Clontech) at the *Nhe*I/*Xho*I sites. The OXTR was subcloned into the pAmCyan1-N1 CFP vector at the *Eco*RI/*Nhe*I sites. Each construct was transiently transfected into HEK293MSR cells using Lipofectamine 2000 (Thermo Fisher Scientific), according to the manufacturer’s instructions.

### FRET

FRET was performed using a confocal laser microscopy as previously described with slight modification [11, 12]. In brief, 1 μg of each expression vector (EP2-fused CFP (EP2-CFP), OXTR-fused CFP (OXTR-CFP), or CTR-fused YFP (CTR-YFP)) was transfected to HEK293MSR cells on the 35-mm glass-bottom dish. CFP, YFP, and FRET signals were visualized at the following day using confocal laser microscopy, Fluoview FV1000 (Olympus, Tokyo, Japan). CFP and YFP were excited with the 458- and 515-nm lines of an argon laser, respectively. The emitted fluorescence was collected at 475–500 nm for CFP and 530–630 nm for YFP and FRET signals. Dose response effects of EP2-expression levels on FRET were investigated as follows. 1 μg of CTR-YFP expression vector was transfected with 0, 0.01, 0.1, 0.5, and 1 μg of EP2-CFP expression vector to 1×10^5^ HEK293MSR cells on the 35-mm glass-bottom dish. After 24 hr incubation, FRET signal was observed. The signal intensity was analyzed using Fiji software and data was represented as % maximal intensity.

### Second messenger detection

Intracellular Ca^2+^ mobilization and cAMP production were measured as previously described [11, 12]. In brief, 1×10^6^ HEK293MSR cells were spread on 60-mm dish at one day before transfection. 10 μg of pcDNA-based vector (5 μg of one GPCR-V5 and 5 μg of another) or mock vector were transfected into the cells. As for the evaluation of dose response effects of EP2-expressioon levels on Ca^2+^ mobilization, 2.5 μg of CTR-V5 expression vector was transfected with 0, 0.5, 1, 2, and 2.5 μg of EP2-V5 expression vector to 5×10^5^ cells on the 35-mm dish. After the 24 hr incubation, 5×10^4^ cells per well were re-spread to the 96-well plate for the following assay. Prostaglandin E2 (PGE2, sc-201225, Santa Cruz Biotechnology) and salmon CT (sc-201167, Santa Cruz Biotechnology) were used for the assays. Real-time fluorescent measurement for Ca^2+^ mobilization using a Ca5 kit (Molecular Devices, Sunnyvale, USA) and end point observation of cAMP production using CatchPoint Cyclic AMP Assay Kit (Molecular Devices) were performed on FlexStation II. Results are shown as means ± standard error of the mean (SEM) of three independent experiments. Emax values between monomer- and heterodimer-expressing cells were analyzed by Student’s *t* test. *P* < 0.05 was defined as statistically significant.

## Results and discussion

Previously, we showed that Ci-GnRHR1 (R1) and Ci-GnRHR4 (R4) formed heterodimers following their transfection into HEK293MSR cells [11, 12]. Therefore, we first assessed whether a known heterodimer between V5-tagged R1 and Myc-tagged R4 (R1_V5_-R4_Myc_) could be detected by Co-IP-based LC-MS/MS analysis, which consists of five steps: 1) purification of membrane proteins, 2) Co-IP, 3) protease digestion, 4) nano-scaled LC-MS/MS, and 5) database assignment (S1 Text). LC-MS/MS analysis of V5-coimmunoprecipitates detected protein fragments of R1 and R4 (S1 and S2 Figs), confirming the suitability of this method for identifying GPCR heterodimers.

**Fig 1:**
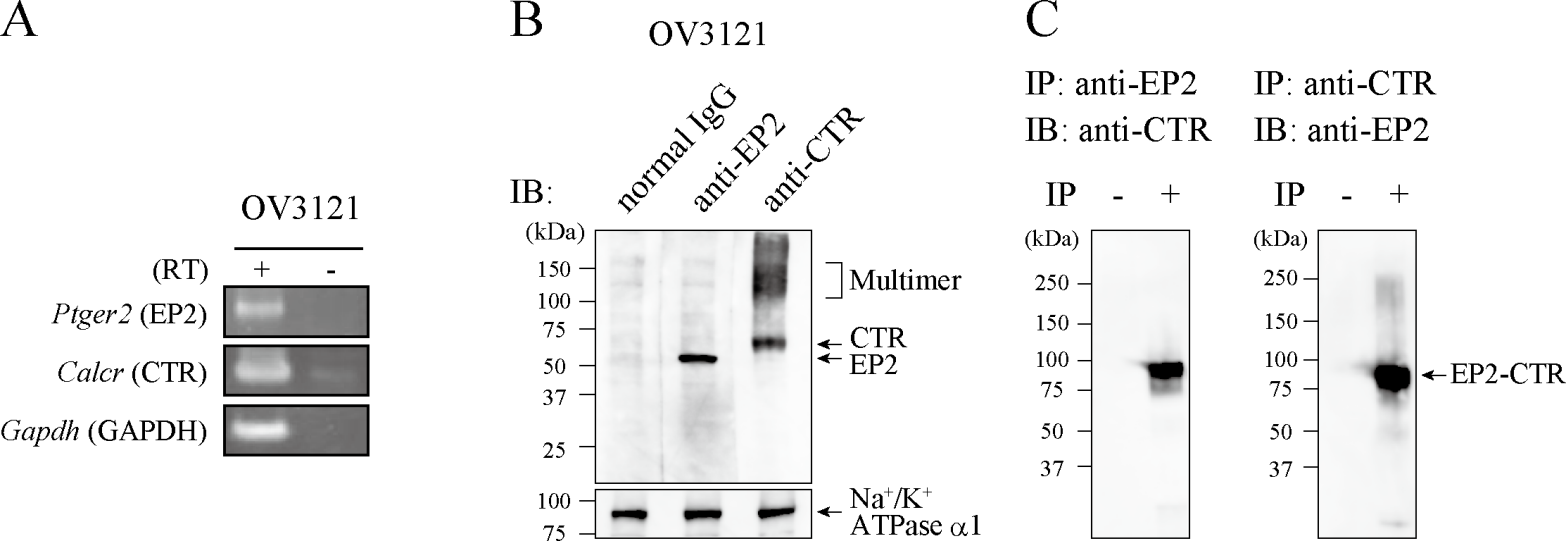
EP2 interacts with CTR in the mouse ovarian granulosa cell line, OV3121. **(A) *Ptger2* and *Calcr* mRNA expression in OV3121 cells.** Total RNA was isolated from OV3121 cells, and first strand cDNA was synthesized with (RT+) or without (RT-) reverse transcriptase, followed by RT-PCR. Gene symbols are indicated in *italic*, and their protein names are presented in parentheses. The *Gapdh* gene was used as an internal control. Abbreviations: *Ptger2* (EP2), prostaglandin E2 receptor 2; *Calc*r (CTR), calcitonin receptor; *Gapdh* (Gapdh), glyceraldehyde-3-phosphate dehydrogenase. **(B) EP2 and CTR expression in OV3121 cells.** Western blotting using rabbit monoclonal anti-EP2 (ab167171) and anti-CTR (ab11042) antibodies detected specific bands for monomeric and/or oligomeric GPCR at the predicted size. 1 μg of normal rabbit IgG (sc-2027) was used as a negative control. A plasma membrane marker, Na^+^/K^+^ ATPase α1 was stained with the antibody (ab7671) as a loading control. **(C) Detection of EP2-CTR interaction.** Western blotting of EP2- (left) and CTR- (right) coimmunoprecipitates using respective anti-CTR (left) and anti-EP2 (right) antibody detected specific bands for EP2-CTR heterodimer. Corresponding amount of sample prepared without Co-IP (IP-) was used as negative control.

Detection of known GPCR heterodimer heterologously expressed in HEK293MSR cells by the Co-IP-based LC-MS/MS method prompted us to identify a novel endogenous GPCR heterodimers in native tissues and cells using this experimental method. First, we investigated the GPCR expression in murine ovarian granulosa cell line, OV3121. RT-PCR analysis showed that PGE2 receptor 2, EP2 mRNA (*Ptger2*) was expressed in OV3121 cells (Fig 1A). Although EP2 was reported to be expressed in native granulosa cells of preovulatory follicles [15–18], whether EP2 forms GPCR heterodimer with other GPCRs and its functional consequences are not known. Subsequently, we utilized the Co-IP-based LC-MS/MS to identify GPCR protomers that heterodimerize with EP2 in OV3121 cells.

To identify endogenous GPCR heterodimers, we optimized three steps of the Co-IP-based LC-MS/MS analysis. First, in purifying membrane proteins, cell lysates were treated with RNase A to remove impurities such as ribosomal proteins and to enhance the solubility of membrane proteins [26]. Second, because rabbit monoclonal antibodies have shown extremely high affinity and specificity [28–30], in the Co-IP step, a rabbit monoclonal anti-EP2 antibody was used. Western blotting with this antibody detected specific band corresponding to EP2, whereas no band was observed using the normal rabbit IgG (Fig 1B, top). These results confirmed the expression of EP2 in OV3121 cells and the specificity of this antibody. Additionally, the expression of Na+/K+ ATPase α1 was indicated as a loading control (Fig 1B, bottom). Third, EP2-coimmunoprecipitates were serially digested with SDC to improve the accessibility of proteases to hydrophobic GPCR [31–33]. The resultant peptide fragments were subjected to nano-LC-Orbitrap MS/MS analysis, followed by database-referencing. These EP2-immunoprecipitates were found to contain various proteins, including some transporters, enzymes, non-GPCR receptors, and G proteins (S2 Table) as observed in proteomic analysis of other membrane proteins [31]. Notably, EP2-coimmunoprecipitates were found to contain fragments of one GPCR, CTR (S3 Fig), suggesting that EP2 and CTR form a heterodimer. The mRNA expression of CTR (*Calcr*) in OV3121 cells was confirmed by RT-PCR (Fig 1A). Western blotting also detected the bands of monomeric/multimeric CTR and verified the specificity of the anti-CTR antibody (Fig 1B). Thus, we examined the interaction between EP2 and CTR by another Co-IP-Western blotting using anti-EP2 and anti-CTR antibodies. The specific band of EP2-CTR heterodimer was detected in EP2-coimmunoprecipitates with anti-CTR antibody at 90 kDa (Fig 1C, left). Likewise, the identical band was also detected in CTR-coimmunoprecipitates with anti-EP2 antibody (Fig 1C, right), which was not observed in the corresponding amount of sample prepared without Co-IP (Fig 1C, IP-). This is consistent with the previous findings that non-obligatory GPCR heterodimers are not dissociated by SDS [34, 35]. These results indicate that EP2 interacts with CTR in OV3121 cells.

**Fig 2:**
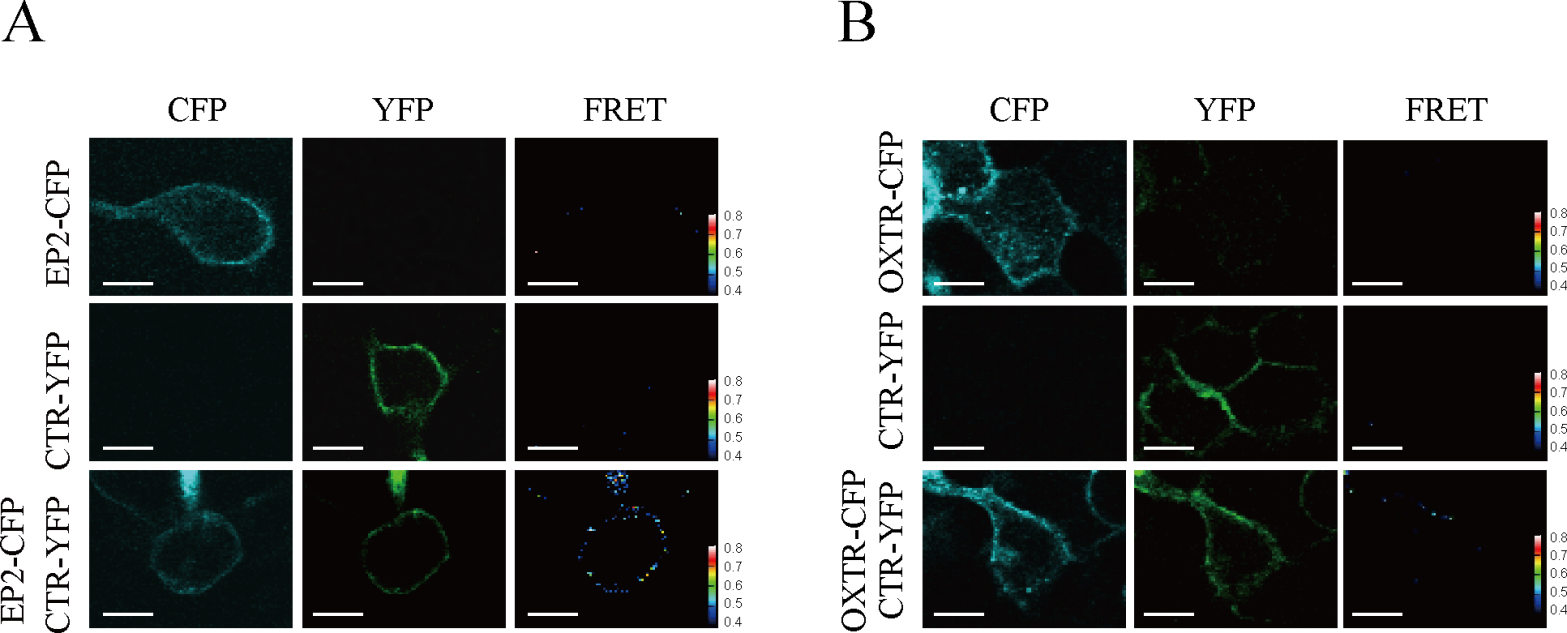
FRET analysis of EP2-CTR heterodimerization. EP2-CFP (A, upper panels) or OXTR-CFP (B, upper panels) and CTR-YFP (middle panels) were expressed individually or coexpressed (lower panels) in HEK293MSR cells. Individual cells were imaged. Left panels, CFP; center panels, YFP; right panels, FRET. Specific FRET signal was detected in EP2-CFP- and CTR-YFP-expressing cells (A), but not in OXTR-CFP- and CTR-YFP-coexpressing cells (B). Color bar indicates the FRET ratio between signal intensities of YFP and CFP. Scale bar represents 10 μm.

To further validate the formation of EP2-CTR heterodimer, FRET analysis was performed. EP2-CFP, CTR-YFP, or both constructs were transfected into HEK293MSR cells. Both CFP- and YFP-derived signals were observed when EP2-CFP and CTR-YFP were expressed alone or together, confirming that both GPCRs were sufficiently expressed on the plasma membranes (Fig 2). Furthermore, prominent FRET signals were detected exclusively in cells coexpressing EP2-CFP and CTR-YFP, but not in cells expressing either alone (Fig 2A). In contrast, no FRET signals were observed in OXTR-CFP- and CTR-YFP-coexpressing cells (Fig 2B), showing that the FRET signals were specific for EP2-CTR (Fig 2A). In addition, FRET signals were dependent on the ratio of EP2-CFP expression to CTR-YFP expression (S4 Fig). Taken together, these findings indicate that EP2 forms specific, constitutive heterodimers with CTR.

Co-IP-based LC-MS/MS analysis has been widely utilized to assess comprehensive interactions of proteins [23, 24, 36–40]. To our knowledge, however, this report is the first to show the identification of a novel GPCR heterodimer by Co-IP-based LC-MS/MS. The ability of this method to detect GPCR protomers is particularly important in improving the specificity and sensitivity of detecting target proteins. In this context, utilizing high-specificity-rabbit monoclonal antibody and stringent detergents (SDS) in the Co-IP step was effective in detecting the specific interaction between EP2 and CTR. Moreover, serial protease-digestion with SDC during fragmentation of coimmunoprecipitates likely enhanced the sensitivity of detection. Multi-epitope affinity purification in a buffer containing detergent of mild to intermediate stringency led to the identification of multiple non-GPCR-transmembrane proteins that form macromolecular complexes with native GPCR [24], suggesting that mixture of several antibodies in a mild stringent detergent may enhance the detection of GPCR heterodimers. Additional enrichment of plasma membrane fractions by density-gradient centrifugation may also remove impurities and enhance the specificity. However, it is noteworthy that a novel GPCR heterodimer, namely, EP2-CTR, was detected by our relatively simple procedure despite the presence of various non-GPCRs and impurities. The results therefore indicate the potential of this Co-IP-based LC-MS/MS method to identify other novel GPCR heterodimers in other cells or even in native tissues.

**Fig 3:**
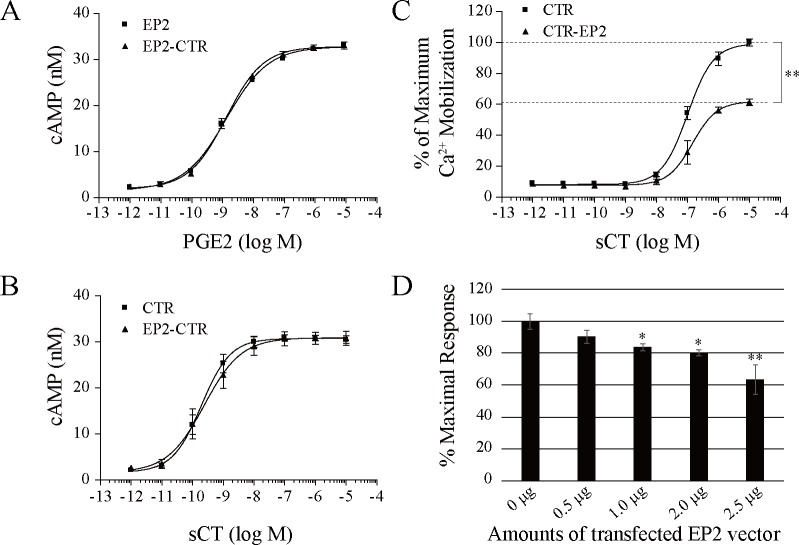
Effect of EP2-CTR heterodimerization on second messenger production. (A-C) 5-μg each of EP2-V5 and CTR-V5 vectors were transfected individually or together into 1×10^6^ HEK293MSR cells on 60-mm dish. Then, 5×10^4^ cells were spread onto 96-well assay plates at the next day and incubated for another 24 hr. cAMP production (A, B) and Ca^2+^ mobilization (C) were measured following stimulation with PGE2 (A) or salmon CT (sCT, B, C) at indicated concentration. Neither PGE2 (A) nor sCT (B) stimulation significantly altered cAMP production by HEK293MSR cells. (C) Ca^2+^ mobilization by ≥10^−7^ M sCT was significantly lower in the EP2-CTR-coexpressing cells than in cells expressing CTR alone. Emax values (dashed lines) between monomer- and heterodimer-expressing cells (100.0% ± 2.5% vs 62.4% ± 5.2%) were analyzed by Student’s *t* test (**, *P* = 0.0004). (D) Dose-response effects of EP2-expression levels on Ca^2+^ mobilization. 2.5 μg of CTR-V5 and 0, 0.5, 1, 2, or 2.5 μg of EP2-V5 expression vectors were transfected into 5×10^5^ HEK293MSR cells on 35-mm dishes. Ca^2+^ mobilization was measured as above following stimulation with 1×10^6^ M sCT. Results are shown as the mean ± SEM from 3 independent transfections.

**Fig 4:**
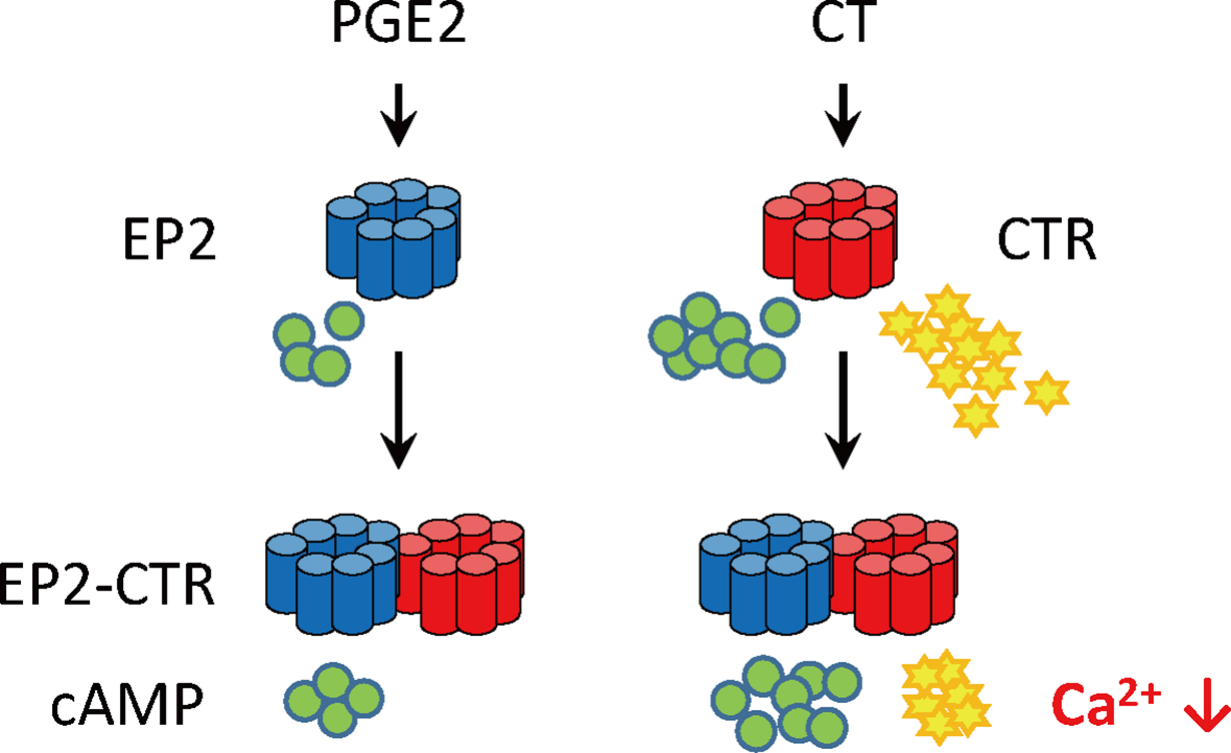
Signaling modulation via EP2-CTR heterodimerization. The EP2-CTR heterodimer suppressed CTR-mediated Ca^2+^ mobilization, compared with the CTR monomer/homodimer. cAMP production inducd by either PGE2 or CT was not affected by heterodimerization.

Subsequently, we evaluated the functional propensities of EP2-CTR in the second messenger production. EP2 is coupled with only Gs protein and elevates the cAMP production upon the PGE2 stimulation [15], whereas CTR are coupled with Gs and Gq proteins and induces both cAMP production and intracellular Ca^2+^ mobilization in response to CT [41]. We thus examined the effect of the heterodimerization on the cAMP production using HEK293MSR cells. Neither CT nor PGE2 stimulation exhibited any significant alteration of cAMP production in heterodimer-expressing cells compared to monomer-expressing cells (Fig 3A and 3B). On the other hand in the intracellular Ca^2+^ mobilization by CT, cells expressing EP2-CTR heterodimer showed significant suppression of intracellular Ca^2+^ mobilization (Emax = 62.4% ± 5.2%, *P* < 0.01, EC_50_ = 1.5 x 10^−7^ M) in a CT dose-dependent fashion, compared with those expressing CTR alone (Emax = 100.0% ± 2.5%, EC_50_ = 1.0 x 10^−7^ M), (Fig 3C). Furthermore, the suppression of Ca^2+^ mobilization was found to be correlated with the amounts of EP2-expression vector (Fig 3D). Taken together, these results, combined with the Co-IP (Fig 1 and S3 Fig) and FRET data (Fig 2 and S4 Fig), strongly indicate that EP2-CTR modulates intracellular CTR-induced Ca^2+^ mobilization in response to CT (Figs 3 and 4), providing evidence for EP2-CTR heterodimerization.

Of particular interest is that heterodimerization of EP2 with CTR exclusively resulted in suppression of intracellular Ca^2+^ mobilization by CTR (Figs 3 and 4), whereas CTR activates not only intracellular calcium mobilization but also cAMP production in response to CT [41]. These results are in good agreement with the findings that GPCRs adopt multiple active conformations which are specific to the respective G proteins and that these active conformations are not interconvertible [3, 42–49]. Furthermore, several protomers of GPCR heterodimers were shown to affect the adoption of a particular active conformation of a partner GPCR as an endogenous allosteric modulator [3, 11, 12, 42–49]. In combination, the present results suggest that EP2 specifically suppresses the adoption of the active conformation of CTR specific to Gq-coupling via EP2-CTR heterodimerization.

In conclusion, we have identified a novel heterodimer, EP2-CTR using a Co-IP-based LC-MS/MS method and demonstrated the regulation of second messenger production in cultured cells. The present study paves the way for investigation of molecular mechanisms underlying a wide range of endocrine, neuroendocrine, and nervous systems involving GPCR heterodimers.

## Supporting information

S1 Text Supplementary materials and methods. (DOCX)

S1 Fig Detection of known R1_V5_-R4_Myc_ heterodimer by Co-IP-based LC-MS/MS. (A) Western blotting using anti-V5 or anti-Myc antibody showed ectopic expression of R1_V5_ or R4_Myc_ in HEK293MSR cells. (B) The amino acid sequences of Ci-GnRHR1 and Ci-GnRHR4 detected by the Co-IP-based LC-MS/MS analysis using anti-V5 antibody are shown in red letters. The sequences of the seven transmembrane-domain are shaded.
(TIF)

S2 Fig Representative MS/MS ion peak patterns of Ci-GnRHR1 and Ci-GnRHR4. b-ion (red) and y-ion (blue) are fragments truncated from C- and N-terminal residues, respectively. Peptide fragments of Ci-GnRHR1 (upper) and Ci-GnRHR4 (lower), corresponding to amino acids 131–148 and 367–382, respectively, were detected. Each fragment was found to include an oxidized methionine residue.
(TIF)

S3 Fig Detection of the EP2-CTR heterodimer by Co-IP-based LC-MS/MS in OV3121 cells. (A) Representative MS/MS ion peak patterns of CTR. b-ion (red) and y-ion (blue) are fragments truncated from C- and N-terminal residues, respectively. Peptide fragments of CTR, corresponding to amino acids 329–340, were detected. (B) Amino acid sequences of CTR detected by the Co-IP-based LC-MS/MS analysis using anti-V5 antibody are shown in red. The sequences of the seven-transmembrane domain are shaded.
(TIF)

S4 Fig Dose-response effects of an EP2-expression vector on FRET. Aliquots of 1-μg CTR-YFP and indicated amounts of EP2-CFP expression vector were transfected into 1×10^5^ HEK293MSR cells on 35-mm glass-bottom dishes. The intensity of the FRET signal observed after 24 hr was analyzed using Fiji software. Data are presented as mean % maximal intensity ± SEM of at least three individual cells.
(TIF)

S1 Table Primer sequences used in the RT-PCR. (DOCX)

S2 Table Protein identification by database-referencing of Co-IP-based LC-MS/MS data. (XLSX)
