# Supplementary material for: Heterodimerization of the prostaglandin E2 receptor EP2 and the calcitonin receptor CTR
Source: PLoS One. 2017 Nov 2;12(11):e0187711. doi: 10.1371/journal.pone.0187711 (PMC5667882; doi:10.1371/journal.pone.0187711)
Supplement: S1 Text — (DOCX) [file pone.0187711.s001.docx]

**Supplementary materials and methods**

**Cell culture and transfection**

HEK293MSR cells stably transfected with V5-tagged Ci-GnRHR1 (R1_V5_) were previously established and maintained under standard conditions [ref. 12 in the main text]. 2×10^6^ cells were spread to 100-mm dish at the day before transfection. 5 μg of Myc-tagged Ci-GnRHR4 (R4_Myc_)-expression vector were transfected in to R1_V5_-expressing cells as described in materials and methods of main text. Transfected cells were harvested at the next day and kept at -80°C until use.

**Co-IP-based MS/MS analysis for R1_V5_-R4_Myc_ detection**

Co-IP-based MS/MS analysis was performed using R1_V5_-R4_Myc_-expressing HEK293MSR cells as described in the materials and methods of main text. Ectopic expression of R1_V5_ or R4_Myc_ was confirmed by Western blotting with anti-V5 or anti-Myc antibody. Co-IP of microsomal fraction of R1_V5_-R4_Myc_-expressing cells was performed using anti-V5 antibody. Immunoprecipitates were digested and analyzed by nano-scale LC Orbitrap Elite MS spectrometry as in the main text. Raw files were processed and referenced to all protein sequences of *Ciona intestinalis* (KH.KHGene.2013Met.protein.zip) downloaded from Ghost Database (http://ghost.zool.kyoto-u.ac.jp/cgi-bin/gb2/gbrowse/kh/).
