# Supplementary figures and images for: Heterodimerization of the prostaglandin E2 receptor EP2 and the calcitonin receptor CTR
Source: PLoS One. 2017 Nov 2;12(11):e0187711. doi: 10.1371/journal.pone.0187711 (PMC5667882; doi:10.1371/journal.pone.0187711)

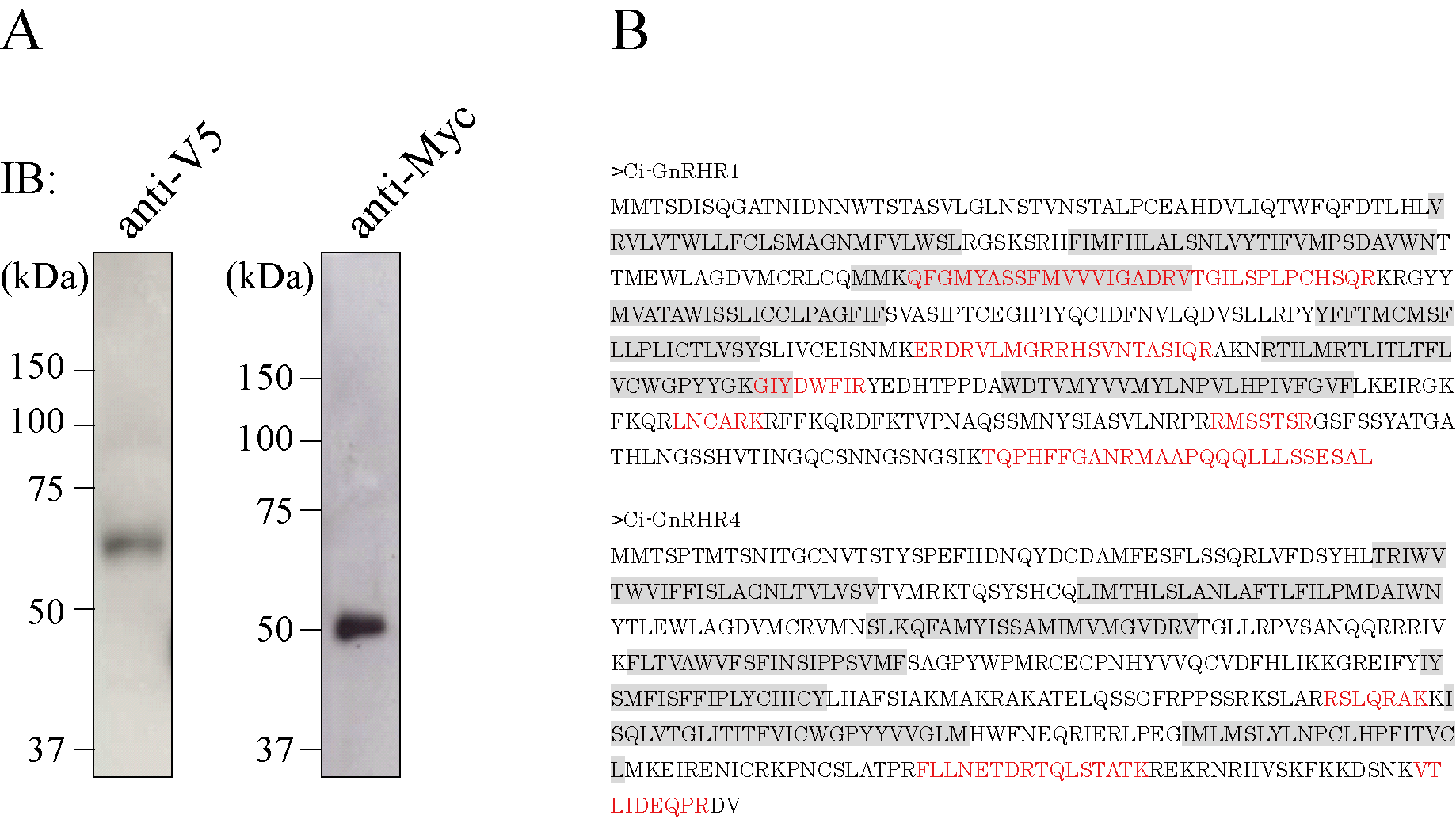

Supplement: S1 Fig — (A) Western blotting using anti-V5 or anti-Myc antibody showed ectopic expression of R1V5 or R4Myc in HEK293MSR cells. (B) The amino acid sequences of Ci-GnRHR1 and Ci-GnRHR4 detected by the Co-IP-based LC-MS/MS analysis using anti-V5 antibody are shown in red letters. The sequences of the seven transmembrane-domain are shaded. (TIF) [file pone.0187711.s002.tif]

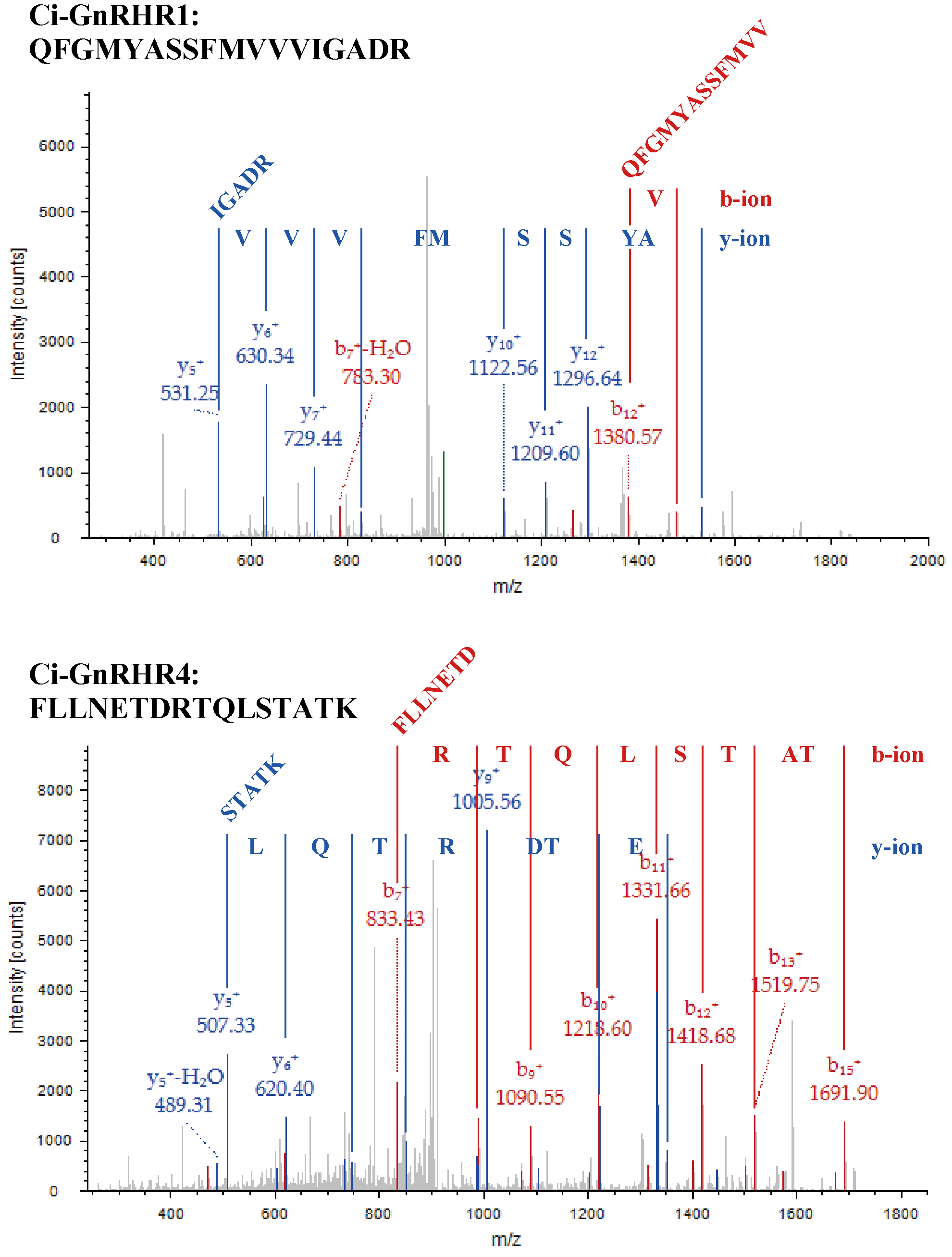

Supplement: S2 Fig — b-ion (red) and y-ion (blue) are fragments truncated from C- and N-terminal residues, respectively. Peptide fragments of Ci-GnRHR1 (upper) and Ci-GnRHR4 (lower), corresponding to amino acids 131–148 and 367–382, respectively, were detected. Each fragment was found to include an oxidized methionine residue. (TIF) [file pone.0187711.s003.tif]

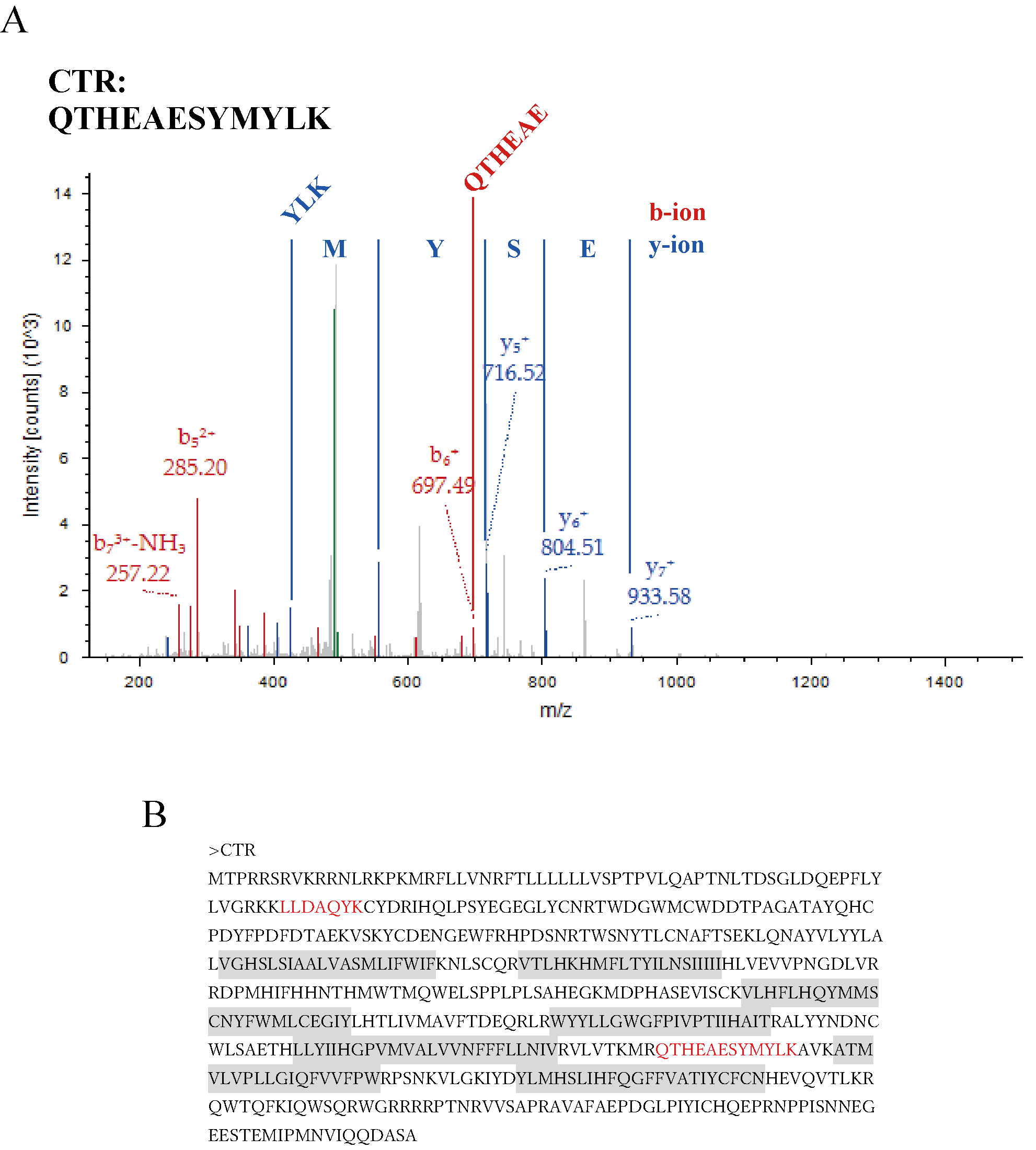

Supplement: S3 Fig — (A) Representative MS/MS ion peak patterns of CTR. b-ion (red) and y-ion (blue) are fragments truncated from C- and N-terminal residues, respectively. Peptide fragments of CTR, corresponding to amino acids 329–340, were detected. (B) Amino acid sequences of CTR detected by the Co-IP-based LC-MS/MS analysis using anti-V5 antibody are shown in red. The sequences of the seven-transmembrane domain are shaded. (TIF) [file pone.0187711.s004.tif]

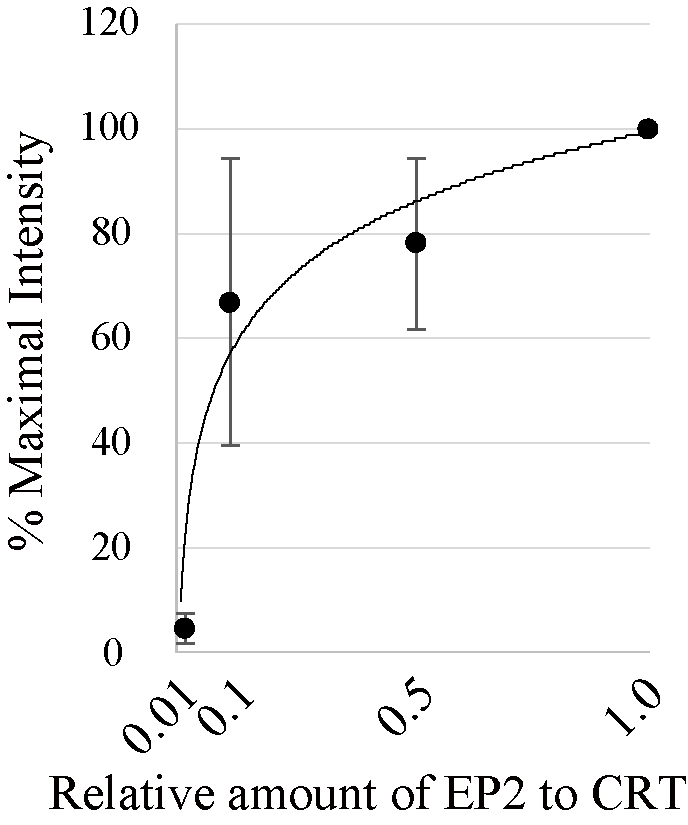

Supplement: S4 Fig — Aliquots of 1-μg CTR-YFP and indicated amounts of EP2-CFP expression vector were transfected into 1×10^5^ HEK293MSR cells on 35-mm glass-bottom dishes. The intensity of the FRET signal observed after 24 hr was analyzed using Fiji software. Data are presented as mean % maximal intensity ± SEM of at least three individual cells. (TIF) [file pone.0187711.s005.tif]
