# Supplementary material for: Heterodimerization of the prostaglandin E2 receptor EP2 and the calcitonin receptor CTR
Source: PLoS One. 2017 Nov 2;12(11):e0187711. doi: 10.1371/journal.pone.0187711 (PMC5667882; doi:10.1371/journal.pone.0187711)
Supplement: S1 Table — (DOCX) [file pone.0187711.s006.docx]

**S1 table. Primer sequences used in the RT-PCR**

| Gene symbol | Forward | Reverse |
| --- | --- | --- |
| *Ptger2* | GTACCCTTACTTCTACAGGC | CCTAAGTATGGCAAAGACCC |
| *Calcr* | GACCGGATTCATCAGTTGCC | GCAATCGACAAGGAGTGACC |
| *Gapdh* | CATGACCACAGTCCATGCCATC | TAGCCCAAGATGCCCTTCAGTG |
